# NOUS-209 Off-the-shelf Immunotherapy Has the Potential to Hit Primary and Metachronous Colorectal and Urothelial Cancers in Lynch Syndrome

**DOI:** 10.1158/1535-7163.MCT-25-0864

**Published:** 2025-11-12

**Authors:** Lorenzo De Marco, Elisa Micarelli, Joni Panula, Jussi Nikkola, Lauri Moilanen, Matti Annala, Jouni Härkönen, Kalle E. Hokkanen, Anna Morena D’Alise, Kirsi Pylvänäinen, Päivi T. Peltomäki, Maarit Ahtiainen, Jan Böhm, Jukka-Pekka Mecklin, Elisa Scarselli, Toni T. Seppälä

**Affiliations:** 1 Nouscom SRL, Rome, Italy.; 2 NEON-S Research Unit, Faculty of Medicine and Health Technology, Tampere University, Tampere, Finland.; 3 Department of Surgery, Vaasa Central Hospital, Vaasa, Finland.; 4 Tays Cancer Centre, Tampere University Hospital, Tampere, Finland.; 5 Department of Urology, Tampere University Hospital, Tampere, Finland.; 6 Department of Pathology, Hospital Nova of Central Finland, Jyväskylä, Finland.; 7 Department of Education and Science, Central Finland Hospital Nova, Wellbeing Services County of Central Finland, Jyväskylä, Finland.; 8 Department of Medical and Clinical Genetics, University of Helsinki, Helsinki, Finland.; 9 HUSLAB Laboratory of Genetics, HUS Diagnostic Center, Helsinki University Hospital, Helsinki, Finland.; 10 Central Finland Biobank, Wellbeing Services County of Central Finland, Jyväskylä, Finland.; 11 Department of Education and Research, Well Being Services County of Central-Finland, Jyväskylä, Finland.; 12 Faculty of Sports and Health Sciences, University of Jyväskylä, Jyväskylä, Finland.; 13 Department of Gastroenterology and Alimentary Tract Surgery, Tampere University Hospital, Wellbeing Services County of Pirkanmaa, Tampere, Finland.; 14 Applied Tumor Genomics, Research Programs Unit, University of Helsinki, Helsinki, Finland.

## Abstract

Lynch syndrome is characterized by the development of microsatellite-instable cancers that share neoantigens, offering an opportunity for targeted immunotherapy. NOUS-209 is a heterologous prime-boost cancer vaccine in clinical development, employing viral vectors encoding 209 shared neoantigen peptides derived from frameshift mutations (FSM) commonly found in microsatellite-instable tumors. In this study, we investigated the presence and dynamics of NOUS-209 targeted FSMs in both primary and metachronous Lynch syndrome–associated cancers. Whole-exome sequencing was performed for 73 tumors, including 58 colorectal cancers and 15 urothelial cancers, from 58 individuals with confirmed Lynch syndrome. A median of 57 FSMs per colorectal cancer and 24 FSMs per urothelial cancer were observed, with similar FSM burdens in both primary and metachronous tumors. Analysis of nine matched primary–metachronous tumor pairs revealed evidence of immune editing: FSMs predicted to encode highly immunogenic neoepitopes were selectively lost in metachronous tumors, whereas those with lower predicted immunogenicity were retained. Importantly, all subsequent primary cancers acquired novel FSMs encoding neoantigens with strong predicted HLA-binding affinity, supporting the rationale for NOUS-209–mediated immune interception. These findings demonstrated that NOUS-209 FSMs are present in both colorectal cancers and urothelial cancers in Lynch syndrome, expanding the therapeutic potential of NOUS-209 beyond colorectal cancer. Moreover, the emergence of novel targetable FSMs in metachronous tumors suggests that NOUS-209 immunotherapy may be effective in the prevention of both primary and metachronous Lynch syndrome–associated cancers.

## Introduction

Lynch syndrome is one of the most common hereditary cancer predisposition conditions caused by a heterozygous germline pathogenic variant in one of the DNA mismatch repair (MMR) genes (*MLH1*, *MSH2*, *MSH6*, or *PMS2*) or by a deletion in *EPCAM*, which induces methylation of the adjacent *MSH2* promoter, thereby impairing MMR function (1). Lynch syndrome confers the most pronounced risk for colorectal and endometrial cancers but also increases the risk for other malignancies, including urothelial cancer (2).

MMR-proficient cells become MMR deficient by acquiring a second hit, often a loss of heterozygosity, in the previously wild-type allele of an MMR gene already carrying a germline pathogenic variant, in accordance with Knudson two-hit hypothesis (3). The MMR system plays a crucial role in maintaining genomic integrity by correcting single-base mismatches and small insertion–deletion loops (indel) that arise during DNA replication. Indels frequently occur in mononucleotide repeats, a type of microsatellite that constitutes a small portion of the genome and is particularly vulnerable to replication errors. The accumulation of mutations in these regions leads to microsatellite instability (MSI; ref. 1). Consequently, indels in mononucleotide repeats are often shared among MSI cancers. Indels occurring in coding regions can generate frameshift peptides (FSP), presented as novel protein sequences—neoantigens—with high immunogenic potential (4). Neoantigens have been extensively studied to determine their ability to elicit spontaneous immune responses and assess their potential in preventing the development of metachronous or subsequent primary tumors. Importantly, shared neoantigens have paved the way for the development of cancer vaccines aimed at prevention or early interception of cancer (5).

Previously, we identified 204 frameshift mutations (FSM) encoding 209 neoantigen peptides common to MSI colorectal cancer, endometrial cancer, and gastric cancer. These FSMs were selected through systematic analysis of MSI tumor exomes from The Cancer Genome Atlas (TCGA), focusing on single-nucleotide deletions within coding mononucleotide repeats. Candidate mutations were prioritized based on their recurrence across MSI tumors, low frequency in normal tissues, and confirmed expression of the mutated peptide (6). The developed NOUS-209 collection has been validated in metastatic cancers predominantly of sporadic origin and has initiated clinical evaluation of product safety and immunogenicity (7, 8).

In pooled analyses of MSI cancers, only a small proportion was attributable to Lynch syndrome, highlighting the need for studies specifically focused on Lynch syndrome–associated cancers. Moreover, the presence of NOUS-209 FSMs in urothelial cancers has not been examined. Here, we performed whole-exome sequencing (WES) of Lynch syndrome–associated primary and metachronous colorectal cancer and urothelial cancer to investigate the presence of NOUS-209 FSMs and their association with clinicopathologic characteristics.

## Materials and Methods

### General statement on scientific rigor adherence

Although this study incorporated patient-derived sequencing data, it was preclinical and experimental in design. Accordingly, certain clinical rigor criteria, such as reporting subject demographics and analysis of sex as a biological variable, were addressed where applicable. Other elements, such as randomization, blinding, attrition, and power analysis, were not relevant to the experimental context.

### Patient cohorts and sample collection

Colorectal cancer and urothelial cancer specimens were retrospectively collected from a cohort of patients diagnosed with Lynch syndrome, identified through the Lynch Syndrome Research Registry of Finland (LSRFi), which includes verified heterozygous pathogenic variant carriers. Formalin-fixed, paraffin-embedded (FFPE) tissue blocks were retrieved via the Finnish Biobank service. Clinical data from LSRFi were cross-referenced with biobank records to select individuals with available colorectal cancer and urothelial cancer specimens, ensuring an accurate linkage between clinical information and corresponding tissue samples.

Histopathologic assessment was conducted to confirm tumor diagnosis and determine key clinicopathologic features, including tumor stage, lymphovascular and perineural invasion, tumor budding, histologic grade for colorectal cancer, and stage and histologic grade for urothelial cancer. Clinical information on patient history and Lynch syndrome gene germline variants was obtained from the LSRFi. Among the 58 colorectal cancer tumor samples, 42% (24 samples) had a matched normal reference sample available for alignment. Of the 15 urothelial cancer samples, 93.3% (14 samples) had a corresponding normal sample.

### IHC

IHC was performed to determine the expression of MLH1, PMS2, MSH2, MSH6, and β2-microglobulin (B2M). Following standard procedures, IHC staining was performed on FFPE sections using the Leica BOND-III automated IHC stainer (RRID: SCR_026521) and BOND Polymer Refine Detection kit (Leica Biosystems, cat. #DS9800, RRID: AB_2891238). Antigen retrieval was performed with EDTA-based buffer, pH 9 (BOND Epitope retrieval solution 2, Leica Biosystems, AR9640), at 100°C for 20 minutes (MSH2 and B2M) or 30 minutes (MLH1, MSH6, and PMS2). The antibody dilutions were 1:50 for MLH1 (Leica Biosystems, cat. #NCL-L-MLH1, RRID: AB_10555424), ready to use (RTU) for MSH2 (Leica Biosystems, cat. #PA0989, RRID: AB_3697735), 1:150 for MSH6 (Abcam, cat. #AC-0047, RRID: AB_11001535), RTU for PMS2 (Leica Biosystems, cat. #PA0991, RRID: AB_2936884), 1:1000 for B2M (Cell Signaling Technology, cat. #12851, RRID: AB_2716551), and 1:200 for CD8 (Leica Biosystems, cat. #CD8-4B11-L-U, RRID: AB_3676740). An incubation time of 60 minutes was used for all antibodies. Normal MLH1, PMS2, MSH2, and MSH6 expression in tumor samples was detected by nuclear staining and B2M by cytoplasmic/membranous staining of neoplastic epithelial cells. Loss of expression was indicated by a lack of expression in tumor cells combined with positive staining of internal positive controls (stromal cells or blood vessels). In the case of B2M, the heterogeneity of staining was considered. CD8^+^ cell densities were calculated by dividing the cell counts by the tumor core area in mm^2^ in tumor core and at invasive margin.

### WES of colorectal cancer

A total of 50 ng of gDNA from FFPE tumor samples was processed using Twist Library Preparation EF 2.0, with Enzymatic Fragmentation and Twist Target Enrichment Protocols (Twist Bioscience). Unique dual-index oligos from Integrated DNA Technologies were used as the ligation adapters. Libraries were pooled into nine- and 10-plex reactions and enriched using Twist Core Exome Refseq Targets (hg38; 37 Mb). Sequencing was performed using the Illumina NovaSeq system with paired-end reads (101 + 8 + 8 + 101 bp) and v1.5.

Tumor samples were aligned to the hg38 reference genome, and variant calling was performed using the Illumina DRAGEN pipeline by the Finnish Institute of Molecular Medicine Genomics NGS Unit at the University of Helsinki. Variant calls were annotated with Ensembl Variant Effect Predictor (v. 110.1, RRID: SCR_007931; ref. 9) and converted to maf format using vcf2maf (v. 1.6.21, RRID: SCR_027063). Variants were initially filtered based on the depth of coverage, allelic fraction (AF), and population allele frequencies from the gnomAD genome and exome databases (gnomAD r3.1.1 and r2.1.1; refs. 10, 11). Variants were included if they passed the quality control filter from the DRAGEN pipeline and had a minimum coverage of 20×. Both the reference and variant alleles required at least six reads for inclusion. Variants in genes with symbols such as orf, ENSG, ENSM, ENST, and “Unknown” were excluded. Only the variants on chromosomes 1 to 22 and X were considered. For tumor-only samples, variants with a population allelic frequency exceeding 1% in both global and Finnish populations from the gnomAD database were excluded. Variants were further refined by their absolute AF, excluding those with an AF below 0.05, and by their mean-normalized AF ratio, with variants showing a zygosity ratio greater than five being removed. Mode-normalized AF was also considered, which required a minimum value of 0.15, and all noncoding variants were excluded. Only variants classified as pathogenic or likely pathogenic in ClinVar (RRID: SCR_006169; ref. 12) and those predicted to result in loss of function (LoF) were included in the final analysis.

### WES of urothelial cancer

Sequencing libraries were prepared and processed using CeGaT, and paired-end sequencing (2 × 101 bp) was performed on an Illumina NovaSeq 6000 platform. Demultiplexing was performed using bcl2fastq (v2.20, RRID: SCR_015058), and adapter sequences were trimmed using Skewer (v0.2.2, RRID: SCR_001151). FASTQ files were analyzed using the Illumina DRAGEN Bio-IT Platform (v4.2.4). Sequencing reads were aligned to the GRCh38/hg38 reference genome and duplicate reads were marked. For samples with matched normal and tumor tissues, somatic variant calling was performed using the DRAGEN somatic pipeline. For tumor-only samples, a tumor-only DRAGEN analysis workflow was used. The variants were annotated and filtered using the same pipeline as that used for the colorectal cancer cohort.

### NOUS-209 FSM detection

NOUS-209 FSMs were identified from the aligned sequencing data (BAM files) using a lookup approach. A mutation was considered present if at least three mutant-supporting reads were detected and the variant allele frequency exceeded 10%, consistent with previously established criteria (6).

### MSI status assessment

The analysis pipeline included indexing of the GRCh38/hg38 reference genome and scanning of 2,793 microsatellite loci. These loci were selected based on their ability to effectively distinguish between microsatellite-stable (MSS) and MSI samples as determined by the MSIsensor2 model trained on TCGA data. MSI scores were then calculated based on the proportion of unstable sites. Samples with MSI scores >20% were classified as MSI, whereas those with scores <20% were considered MSS. All analyses were performed using the default parameters of MSIsensor2.

### HLA typing and MHC class I and II epitope predictions

High-resolution HLA class I typing (HLA-A, -B, and -C) was performed by aligning raw FASTQ files to the HLA reference genome using BWA-MEM (v0.7.17, RRID: SCR_010910; ref. 13), followed by analysis with OptiTypePipeline (v1.3.1, RRID: SCR_022279; ref. 14). MHC class I peptide binding affinity predictions were conducted using the Immune Epitope Database and Analysis Resource MHC class I (MHC-I) prediction tools (RRID: SCR_006604). The peptide sequences of interest were evaluated using the consensus method (v2.18; ref. 15), focusing on nonamer (nine-mer) peptides. Peptides with predicted IC_50_ below 500 nmol/L were classified as strong binders.

High-resolution HLA class II typing was performed using PHLAT (16). MHC class II peptide binding affinity predictions were conducted with NetMHCIIpan (v4.2). The peptide sequences of interest were evaluated across multiple lengths (from 12 amino acids to 16 amino acids). Peptides with predicted IC_50_ below 500 nmol/L were classified as strong binders.

### Statistical analysis and data visualization

All statistical analyses and data visualizations were conducted using R software (v. 4.4.2, RRID: SCR_001905). Group comparisons of continuous variables were conducted using the Wilcoxon rank-sum test, whereas categorical variables were compared using the Fisher exact test. Correlation analyses were performed using Pearson correlation coefficient. All tests were two-tailed, and *P*-values < 0.05 were considered statistically significant. Analyses were performed using the Stats package (v. 4.6.0, RRID: SCR_025968) in R.

### Ethical considerations

All phases of the study were conducted in accordance with the Declaration of Helsinki and approved by the institutional review board. The LSRFi is based on both consent-waived secondary use of health data and consent-based protocols. Based on the biobank legislation, all contributing patients gave their consent to the biobank for their data and specimens to be used in research, which were separately reviewed by each biobank before material transfer to researchers. All data were pseudonymized prior to the analysis to protect patient privacy. The following ethical and biobank approvals were used for the sample and data acquisition: KSSHP February 15, 2013, KSSHP February 23, 2018, Ethics Committee of KSSHP Dro 466/E6/01, Findata THL/614/14.06.00/2022, National Supervisory Authority of Health and Welfare 10741/06.01.03.01/2015, and Finnish Biobank BB2021-0208. Central Finland Wellbeing Services County Institutional Review Board approved the study. Need to obtain informed consent was waived (Valvira 10741/06.01.03.01/2015).

This study did not involve the use of animals. Accordingly, approval from an institutional animal ethics committee was not required.

## Results

### Clinical characteristics of the cohorts

To evaluate the potential of NOUS-209 in intercepting Lynch syndrome–associated cancers, 73 tumors from 58 patients enrolled in a surveillance program were analyzed. The cohort included 58 patients with colorectal cancer and 15 with urothelial cancer. MMR status was determined in all cases by IHC. All tumors demonstrated MMR protein loss, which was consistent with the germline variant previously identified in each patient, confirming MMR deficiency.

In the colorectal cancer cohort, 38 tumors (66%) were classified as primary and 20 (34%) as metachronous. The mean age at diagnosis was 46.1 years (±12.4 years) for primary colorectal cancers and 61.9 years (±13.5 years) for metachronous cases. *MLH1* (87.5%, *n* = 51) was the predominant MMR germline variant, followed by *MSH2* (12.5%, *n* = 7). The tumor location was predominantly right-sided (60.3% from the cecal to the transverse colon), with 25.9% located on the left side (from the splenic flexure to the sigmoid colon) and 8.6% in the rectum. The stage distribution was comparable between primary and metachronous colorectal cancers, with stage I being the most common (57.9% in primary vs. 55.0% in metachronous), followed by stage II (28.9% vs. 30.0%) and stage III (13.2% vs. 15.0%; Table 1).

**Table 1.** Clinical characteristics of the colorectal cancer and urothelial cancer cohorts.

Characteristic	CRC (*n* = 58)	UC (*n* = 15)
Age, years (mean ± SD)	51.6 ± 14.8	65.8 ± 7.2
Sex	​	​
Female	25 (43.1%)	7 (46.7%)
Male	33 (56.9%)	8 (53.3%)
MMR germline variant	​	​
MLH1	51 (87.9%)	6 (40%)
MSH2	7 (12.1%)	7 (46.7%)
MSH6	0 (0%)	2 (13.3%)
Chronicity	​	​
Primary	38 (65.5%)	13 (86.7%)
Metachronous	20 (34.5%)	2 (13.3%)
Location	​	​
Right	35 (60.3%)	N/A
Left	15 (25.9%)	N/A
Rectum	5 (8.6%)	N/A
Upper urinary tract	N/A	10 (66.7%)
Bladder	N/A	5 (33.3%)
Unknown	3 (5.2%)	0 (0%)
Stage	​	​
I	33 (56.9%)	N/A
II	17 (29.3%)	N/A
III	7 (12.1%)	N/A
IV	0 (0%)	N/A
Non–MI	N/A	8 (53.3%)
MI	N/A	7 (46.7%)
Unknown	1 (1.7%)	0 (0%)
Grade	​	​
Low grade	21 (36.2%)	0 (0%)
High grade	35 (60.3%)	15 (100%)
Unknown	2 (3.4%)	0 (0%)
Budding	​	​
Low	54 (93.1%)	N/A
Intermediate	3 (5.2%)	N/A
Unknown	1 (1.7%)	N/A
Lymphovascular invasion	​	​
No	34 (58.6%)	N/A
Yes	21 (36.2%)	N/A
Unknown	3 (5.2%)	N/A
Extramural vascular invasion	​	​
No	55 (94.8%)	N/A
Yes	1 (1.7%)	N/A
Unknown	2 (3.4%)	N/A
Perineural invasion	​	​
No	56 (96.6%)	N/A
Unknown	2 (3.4%)	N/A
Concurrent carcinoma *in situ*	​	​
No	N/A	8 (53.3%)
Yes	N/A	7 (46.7%)

Abbreviation: CRC, colorectal cancer; UC, urothelial cancer; MI, muscle invasive; N/A, not applicable.

In the urothelial cancer cohort, *MSH2* was the most frequent MMR germline variant (46.7%, *n* = 7), followed by *MLH1* (40.0%, *n* = 6) and *MSH6* (13.3%, *n* = 2). Most urothelial cancers were primary tumors (13 of 15 tumors, 87%), with the remaining two classified as metachronous. Two thirds of urothelial cancers were located in the upper urinary tract and one third in the bladder. Among the primary urothelial cancers, 46.2% were non–muscle invasive (Tis-T1) and 53.8% were muscle invasive (MI; T2–T4); both metachronous tumors were non–muscle invasive. Only one urothelial cancer, classified as primary, exhibited lymph node metastasis (Table 1).

### NOUS-209 FSMs are present in Lynch syndrome–associated colorectal cancers and urothelial cancers

The presence of FSMs targeted by NOUS-209 vaccine was assessed in all tumor samples. Raw WES data were aligned to the human reference genome (GRCh38/hg38) using a multi-step mapping procedure. A previously validated “look-up” analysis was applied to tumor next-generation sequencing data to profile mutations at 31,564 mononucleotide repeat loci located within the open reading frames of coding genes (6).

**Figure 1.**
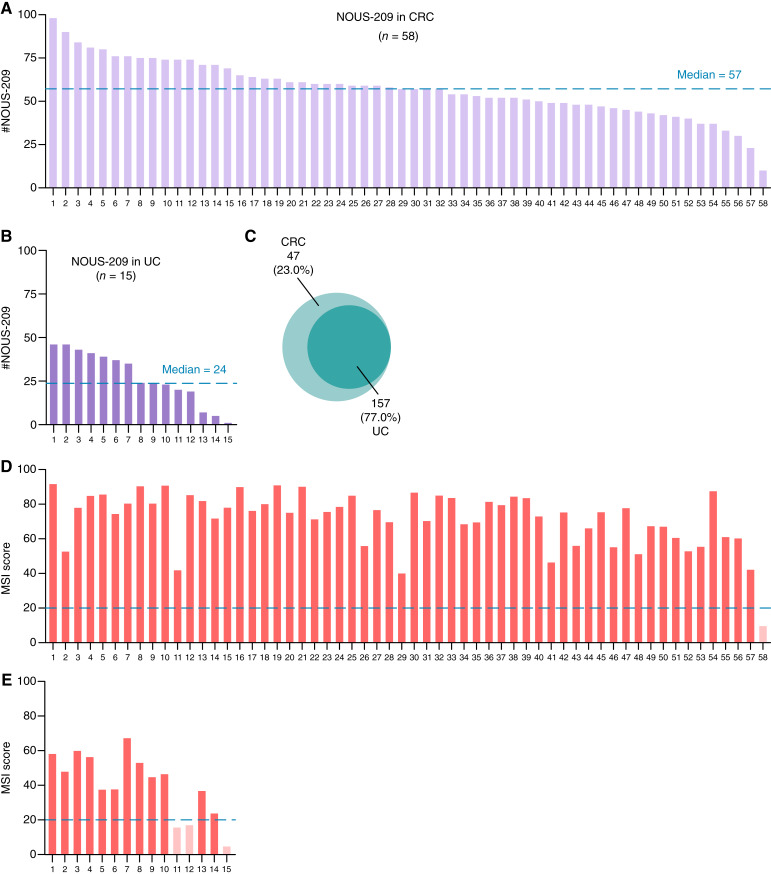
Overview of colorectal cancer and urothelial cancer cohorts. **A** and **B,** Bar plots showing the number of NOUS-209 FSMs detected in each sample of colorectal cancer and urothelial cancer cohorts. The median number of mutations is indicated by the blue dashed line (colorectal cancer = 57; urothelial cancer = 24). **C,** Venn diagram illustrating the overlap of NOUS-209 FSMs between colorectal cancer and urothelial cancer. **D** and **E,** Bar plots displaying the MSI scores in the colorectal cancer and urothelial cancer cohorts, respectively. The blue dashed line represents the recommended threshold of MSI score (20%) for classifying a sample as MSI. Lighter bars indicate samples classified as MSS. CRC, colorectal cancer; UC, urothelial cancer.

Colorectal tumors harbored a median of 57 FSMs per tumor, consistent with previous findings that reported a median of 51 FSMs in a largely sporadic cohort of metastatic colorectal cancers (6). In contrast, urothelial tumors exhibited a lower median of 24 FSMs per tumor (Fig. 1A and B; Supplementary Fig. S1), reflecting the comparatively lower tumor mutational burden (TMB) observed in these samples.

Despite this difference, 157 of the 204 FSMs identified across the cohort (77%) were shared between colorectal cancers and urothelial cancer tumors, underscoring the substantial overlap in the underlying mutational landscapes (Fig. 1C). To better understand the variability in the number of NOUS-209–targeted FSMs among patients, we assessed MSI using MSIsensor2, a tool previously validated for MSI detection (17). This analysis identified one colorectal cancer and three urothelial cancer samples with MSI scores below 20%, which did not meet the threshold for classification as MSI and were therefore considered MSS (Fig. 1D and E). The MSS colorectal cancer sample had the lowest FSM count in the cohort, with only 10 FSMs. Among the MSS urothelial cancer samples, one tumor contained a single FSM, whereas the other two harbored 19 and 20 FSMs, respectively, which was comparable with the FSM burden observed in MSI-classified tumors.

### The median number of NOUS-209 FSMs is comparable across the first, second, and third cancers occurring in Lynch syndrome carriers

**Figure 2.**
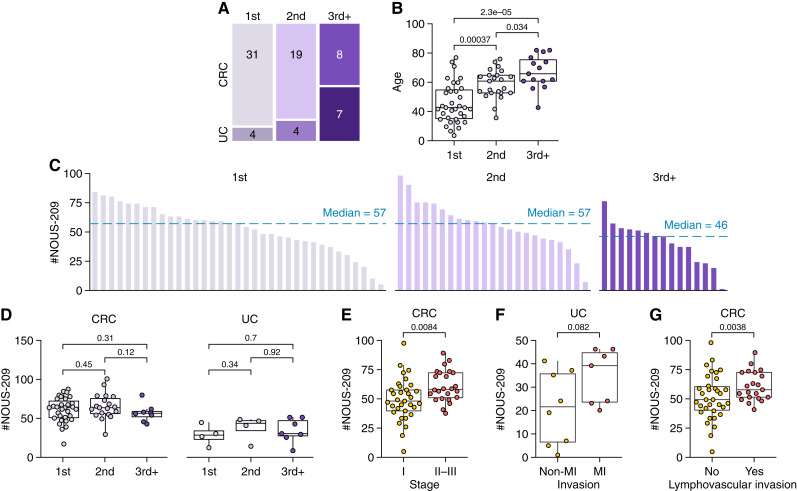
Association of NOUS-209 FSMs with cancer chronology, stage, and invasion. **A,** Mosaic plot showing the distribution of colorectal cancer and urothelial cancer across three cancer groups: first cancer (1st), second cancer (2nd), and third cancer or subsequent cancers (3rd+). **B,** Boxplot of age distribution across the three cancer groups. **C,** Bar plots show the distribution of NOUS-209 FSMs across the three cancer groups. **D,** Boxplots depict the distribution of NOUS-209 FSMs across the three cancer groups and by cancer type. **E** and **F,** Boxplots showing the distribution of NOUS-209 FSMs across stages in the colorectal cancer and muscle invasiveness in urothelial cancer cohorts, respectively. **G,** Boxplots showing the distribution of NOUS-209 FSMs in the colorectal cancer cohort stratified by the presence or absence of lymphovascular invasion. CRC, colorectal cancer; UC, urothelial cancer.

To investigate whether the burden of NOUS-209 FSMs varies with cancer chronology, tumor samples were stratified into three groups based on the sequence of MSI cancer diagnoses for each patient, as recorded in the LSRFi and electronic health records: first, second, and third or subsequent cancer (third^+^). The distribution was as follows: 35 tumors in the first group (31 colorectal cancer and four urothelial cancer), 23 in the second group (19 colorectal cancer and four urothelial cancer), and 15 in the third^+^ group (eight colorectal cancer and seven urothelial cancer; Fig. 2A).

A significant association was observed between cancer type and chronologic grouping, with the third^+^ group demonstrating a higher proportion of urothelial cancer than the first and second groups. This trend likely reflects the natural history of Lynch syndrome–associated urothelial cancer, which generally develops later in life and rarely presents as the first malignancy in patients with Lynch syndrome (2). Consistently, patients in third^+^ were older than those in first and second groups, further supporting age as a contributing factor to the increased prevalence of urothelial cancer in later tumor presentations (Fig. 2B).

NOUS-209 FSMs were identified in tumors across all chronologic groups, with a median of 57 FSMs in both the first group and second group and a slightly lower median of 46 FSMs in the third^+^ group (Fig. 2C). When stratified by tumor type, no significant differences in the FSM burden were observed between the groups, suggesting that the order of tumor occurrence does not explain the variability in the number of targetable mutations (Fig. 2D).

In both the colorectal cancer and urothelial cancer cohorts, a positive correlation was observed between tumor invasiveness and the NOUS-209 FSM burden. Later-stage colorectal cancers (stage II–III compared with stage I) exhibited higher numbers of FSMs, (Fig. 2E). A similar trend was observed in MI urothelial cancer, without statistical significance (Fig. 2F). In colorectal cancer, lymphovascular invasion was also associated with increased FSM counts (Fig. 2G). Altogether, this suggests a potential link between the NOUS-209 FSM burden and disease progression.

### Somatic MMR mutations are frequently observed in Lynch syndrome–associated cancers

To complement the analysis of NOUS-209 FSMs, we examined additional genetic alterations that may contribute to the mutational landscape of Lynch syndrome–associated cancers. Specifically, we focused on pathogenic single-nucleotide variants and FSMs predicted to cause LoF in genes involved in the MMR and genomic stability (base excision repair and nucleotide excision repair) pathways in the Kyoto Encyclopedia of Genes and Genomes (Supplementary Fig. S2). These included *MSH3* and *MSH6*, core components of the MMR pathway (18), as well as *POLD1* and *POLE*, exonuclease domain–containing DNA polymerases essential for replication proofreading, which are known to drive hypermutated phenotypes when defective (19).

**Figure 3.**
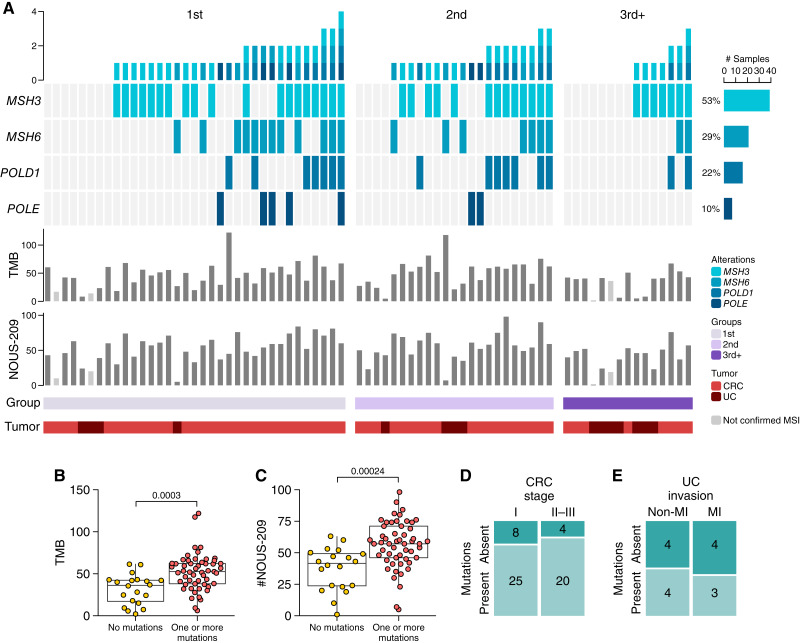
Lynch syndrome–associated cancers frequently carry additional alterations in MMR- and genomic stability–associated genes. **A,** Oncoplot showing the mutational landscape across tumors, classified into three groups based on cancer chronology. Genes are ordered by decreasing mutation frequency. Annotations include TMB (Mut/Mb), the number of NOUS-209 FSMs, and tumor type. In the TMB and NOUS-209 annotation bars, lighter shades indicate samples not confirmed as MSI. **B** and **C,** Boxplots depict the distribution of TMB and NOUS-209 FSM in samples without mutations and in those with at least one mutation in *POLD1*, *POLE*, *MSH3*, and *MSH6* genes. **D** and **E,** Mosaic plot showing the distribution of tumor stage for colorectal cancer and muscle invasion for urothelial cancer, stratified by the presence (at least one mutation) or absence of mutations of no mutation or at least one mutation in *POLE*, *POLD1*, *MSH3*, or *MSH6* genes. CRC, colorectal cancer; UC, urothelial cancer.

Our analysis revealed a high prevalence of pathogenic alterations across these genes: 53% of the tumors harbored mutations in *MSH3*, 29% in *MSH6*, 22% in *POLD1*, and 10% in *POLE* (Fig. 3A). In total, 72.6% of the analyzed tumors carried at least one pathogenic mutation in this gene set. Tumors with at least one such mutation exhibited significantly higher TMB and an increased number of NOUS-209 FSMs compared with tumors without these alterations (Fig. 3B and C). Despite the association between these mutations and elevated TMB, no significant correlation was found between the presence of pathogenic alterations in *MSH3*, *MSH6*, *POLD1*, or *POLE* and advanced tumor stage in colorectal cancer or muscle invasion in urothelial cancer (Fig. 3D and E).

### *B2M* mutations are present in tumors with somatic MMR mutations but are not associated with complete loss of B2M protein expression

**Figure 4.**
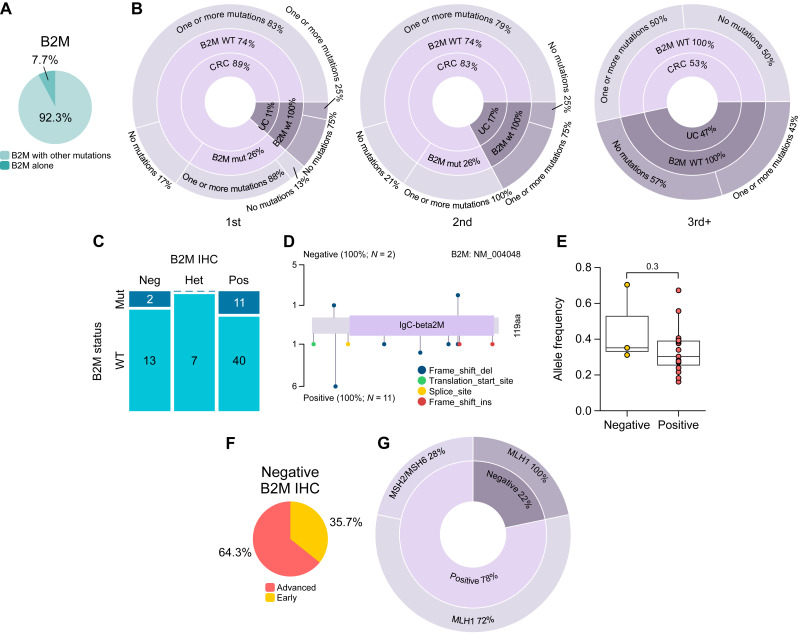
*B2M*-mutated tumors frequently harbor somatic MMR or genomic stability mutations. **A,** Pie chart showing the distribution of *B2M* mutations occurring alone or in combination with MMR (*MSH3* and *MSH6*) or genomic stability (*POLE* and *POLD1*) mutations. **B,** Sunburst charts illustrating the hierarchical distribution of tumors within each group, stratified by *B2M* mutation status (mutant or wild-type) and the presence or absence of at least one MMR or genomic stability mutation. **C,** Mosaic plot showing the distribution of IHC results (Het, heterogeneous) stratified by *B2M* mutation status. **D,** Lollipop plot of *B2M* mutations in samples stratified by IHC status. The plot highlights the distribution and types of somatic mutations along the B2M protein in B2M-negative and B2M-positive cases. **E,** Box plot showing the allele frequency of *B2M* mutations grouped by B2M IHC. **F,** Pie chart showing the distribution of tumor stages in B2M-negative colorectal cancer and urothelial cancer. Early stage was defined as stage I for colorectal cancer and non-MI for urothelial cancer, whereas advanced stage was defined as stage II–III for colorectal cancer and MI for urothelial cancer. **G,** Pie donut chart illustrating the distribution of B2M IHC status (inner ring) and corresponding germline mutations (outer ring). CRC, colorectal cancer; UC, urothelial cancer; WT, wild type.

Given the critical role of B2M in antigen presentation via the MHC-I pathway and its established association with immune escape in MSI tumors (20, 21), *B2M* mutations were specifically investigated across the cohort. Mutations in *B2M* were identified in a subset of tumors and, strikingly, were almost exclusively observed in combination with pathogenic alterations in other genes involved in MMR (*MSH3* and *MSH6*) or genomic stability (*POLE* and *POLD1*). Indeed, in more than 92% of *B2M*-mutated cases, at least one additional mutation in these gene categories was also present (Fig. 4A). Interestingly, *B2M* mutations were completely absent in all urothelial cancer samples and were not detected in any third or subsequent cancers (Fig. 4B).

B2M protein expression was assessed by IHC. A complete loss of B2M staining was observed in 12 colorectal cancers and three urothelial cancers. Surprisingly, no consistent association was found between *B2M* mutation status and protein expression. A substantial number of tumors (*n* = 11) harboring pathogenic or putative LoF mutations retained detectable B2M protein expression, suggesting that many of these variants do not result in complete protein inactivation or may be functionally compensated.

Conversely, in some cases with a complete absence of B2M protein expression, no underlying *B2M* mutation could be identified, suggesting alternative mechanisms, such as epigenetic silencing, posttranscriptional regulation, or other factors influencing protein expression (Fig. 4C). Notably, some mutations were present in both B2M-positive and B2M-negative tumors, indicating that their presence alone is not predictive of the protein expression status (Fig. 4D). Furthermore, no significant difference was observed in the variant allele frequency of pathogenic *B2M* mutations between tumors with and without B2M protein expression, suggesting that clonal abundance does not explain the discrepancies (Fig. 4E).

Interestingly, B2M-negative tumors were more frequently associated with advanced stages (II–III) of colorectal cancer and MI in patients with urothelial cancer (Fig. 4F). Moreover, all B2M-negative tumors were observed in patients with germline *MLH1* mutations (Fig. 4G).

### Immune pressure and the potential of NOUS-209 to intercept subsequent cancers

First, to confirm the presence of relevant neoantigens in the NOUS-209 collection, we looked at correlation between the presence of CD8^+^ cell infiltrate in the tumor and the number of NOUS-209 mutations and TMB in colorectal cancer tumors proficient for antigen presentation (B2M positive by IHC; ref. 22). Interestingly, the presence of the infiltrates in tumor core correlated with the number of NOUS-209 but not with TMB. Although considering CD8^+^ T-cell infiltrates at the invasive margin, no statistically significant association was observed with either NOUS-209 mutations or TMB (Supplementary Fig. S3).

**Figure 5.**
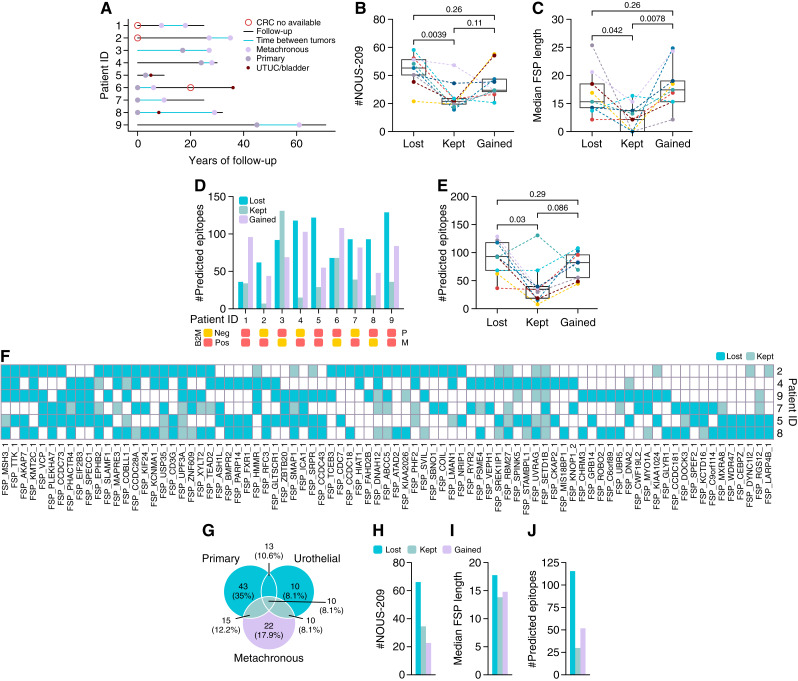
NOUS-209 FSMs lost, gained, and kept between paired tumors. **A,** Timeline plot illustrating the follow-up of patients. Filled dots indicate primary and metachronous colorectal cancer and urothelial cancer. Empty circles denote colorectal cancer for which a tumor sample is not available. Light blue lines indicate the time interval between two tumor occurrences. **B,** Box plot showing, for each patient, the number of NOUS-209 FSMs lost between the first and second tumor, those kept across both tumors, and those gained in the second tumor. **C,** Box plot showing, for each patient, the median length of NOUS-209 FSMs lost, kept, and gained. **D,** Bar plot showing for each patient the number of MHC-I–predicted binders (IC_50_ < 500 nmol/L) for lost, kept, and gained FSMs, with annotations for B2M IHC results below for primary (P) and metachronous (M) samples. **E,** Box plots showing, for each patient, the number of predicted epitopes of NOUS-209 FSM lost, kept, and gained. **F,** Heatmap showing the FSMs lost for each patient, for whom immune editing is hypothesized. FSMs present in the patient's sample are colored if lost or kept. **G,** Venn diagram showing the overlap of mutations across the three tumors from patient Pt8. **H–J,** Line plots for patient Pt8 showing the number of NOUS-209 FSM (**H**), their median length (**I**), and the number of predicted good binders (**J**) across the lost, kept, and gained categories. CRC, colorectal cancer; UC, urothelial cancer.

To specifically investigate neoantigen evolution under immune pressure, we analyzed patients with at least two available MSI tumors. Nine patients with sequential colorectal cancer or urothelial cancer were included in this study. The interval between diagnoses ranged from 2 to 29 years (median: 8.5 years; Fig. 5A). In five cases, additional tumors occurred but were unavailable for sequencing. One patient had three tumors available for sequencing. To assess changes in NOUS-209 FSMs over time, tumor pairs were compared, and FSMs were classified as (i) kept (present in both tumors), (ii) lost (present only in the first tumor), or (iii) gained (present only in the second tumor).

More FSMs were lost than kept in nearly all patients. These lost FSMs tended to encode longer peptides and generated a greater number of predicted CD8^+^ T-cell epitopes with a high binding affinity for HLA class I alleles (IC_50_ ≤ 500 nmol/L; Fig. 5B–E; Supplementary Fig. S4). In contrast, the “kept” FSMs were associated with fewer strong-binding epitopes. These findings suggest that highly immunogenic neoantigens, particularly those producing longer peptides and strong MHC-I binders, are more likely to be absent in subsequent tumors, which is consistent with immune selection pressure and immunoediting.

This immunoediting pattern was observed in nearly all patients, except for three cases (Pt1, Pt3, and Pt6). Notably, in two of these exceptions, the second tumor showed a loss of B2M expression, supporting the hypothesis that when MHC-I–mediated antigen presentation is impaired, previously eliminated neoantigens may reemerge in the tumor.

Interestingly, a very similar pattern to that observed for class-I was also detected when analyzing class-II–predicted epitopes according to class-II typing of each patient with consecutive tumors (Supplementary Fig. S5). These data suggest immunoediting occurring consistently across both class-I and class-II epitopes.

To further explore the role of HLA class I specificity in immune-driven neoantigen loss, we analyzed patients showing clear signs of immunoediting. For each patient, we identified FSMs lost in the second tumor and then focused on those that were recurrently lost across multiple patients (Fig. 5F). We investigated whether patients who lost the same FSMs shared specific HLA class I alleles. In particular, we focused on the two most common alleles, HLA-A02:01 and HLA-A03:01, which were found in three patients. This integrative analysis identified a subset of NOUS-209 FSMs that was recurrently lost in at least two of the three HLA-sharing patients and demonstrated strong *in silico* HLA-binding predictions (Supplementary Table S1).

In all cases, newly arising tumors consistently acquired novel mutations included in the NOUS-209 peptide collection. The median number of gained mutations was 32 (range, 20–59), with a median of 75.5 predicted high-affinity CD8^+^ binders (range, 44–108) and a median peptide length of 18 amino acids (range, 8–170). Importantly, the immunologic features of the gained mutations were comparable with those of lost mutations in terms of peptide length and the number of predicted CD8^+^ epitopes (Fig. 5B–E). These data suggest that despite immunoediting, vaccine efficacy may be preserved owing to the continuous emergence of new, targetable FSMs.

Finally, in one patient (Pt8) with three sequenced tumors, FSMs from the first two tumors were considered lost if they were present in either the first or second tumor but absent in the third. Mutations present in the first and/or second tumor and detected in the third tumor were considered kept, whereas mutations unique to the third tumor were classified as gained (Fig. 5G). Again, lost FSMs were more numerous than kept, with longer peptide lengths and more predicted epitopes (Fig. 5H and I). Gained mutations showed a higher number of predicted CD8^+^ binders than the kept mutations (Fig. 5J). These findings further support the concept that although immunoediting shapes the neoantigen landscape of successive tumors, the ongoing appearance of novel immunogenic FSMs sustains the potential of the NOUS-209 vaccine for immune-mediated tumor interception in Lynch syndrome.

## Discussion

In this study, we investigated the potential of a novel immunotherapy, NOUS-209, currently in clinical development (NCT04041310 and NCT05078866), to intercept cancer in Lynch syndrome carriers. We demonstrated that NOUS-209 FSMs provide broad neoantigen coverage not only in primary colorectal cancers and urothelial cancers but also in metachronous tumors, highlighting its potential as an immune interception strategy for Lynch syndrome–associated cancers. Previous studies have predicted neoantigens using large datasets, including TCGA, focusing mainly on sporadic cancers (4, 23) and Lynch syndrome cohorts (17). Our study stands out in its inclusion of a substantial proportion of metachronous colorectal cancers, enabling—for the first time—the assessment of neoantigen-based strategies aimed at preventing subsequent cancers. Additionally, the potential of cancer vaccines for Lynch syndrome–associated urothelial cancer has not been explored previously.

Colorectal cancers harbored a median of 57 FSMs of 209 FSM-derived FSPs encoded by the vaccine, which is consistent with our earlier findings of a median of 51 FSM-derived FSPs in metastatic colorectal cancers (6). Urothelial cancers exhibited a lower median of 24 FSMs per tumor, reflecting their generally lower TMB compared with colorectal cancer samples. This difference likely reflects intrinsic biological distinctions between tissue types, including variations in the histologic architecture and proliferative dynamics. The colorectal epithelium undergoes rapid turnover, increasing opportunities for replication-associated errors and the resultant FSMs. Conversely, the urothelial epithelium has a lower proliferative index, which potentially limits the accumulation of mutations over time (24, 25). Nevertheless, the presence of a substantial number of NOUS-209 FSMs in urothelial cancers supports the potential of this vaccine to prevent this type of cancer. Importantly, although urothelial cancer is less common than colorectal cancer in Lynch syndrome, it is associated with worse prognosis (26), partly because of disparities in surveillance strategies. Colonoscopy surveillance is universally recommended for Lynch syndrome carriers and might improve survival after colorectal cancer despite its inability to reduce incidence (27). In contrast, there is insufficient evidence to support routine urothelial cancer surveillance in Lynch syndrome, possibly resulting in more advanced-stage diagnoses (28).

Among urothelial cancer cases, 20% of the tumors were classified as MSS by MSIsensor2, whereas only one colorectal cancer (∼2%) was classified as MSS. Interestingly, two of the three MSS urothelial cancers showed a reasonable number of NOUS-209 FSMs. If validated in larger cohorts, this observation supports the potential utility of NOUS-209 even in the early stages of tumorigenesis prior to the full molecular manifestation of the MSI phenotype.

The FSM burden was positively associated with advanced cancer stages, including stage II to III colorectal cancers and MI urothelial cancers, and with high-risk features such as lymphovascular invasion in colorectal cancer, suggesting an accumulation of immunogenic mutations during carcinogenesis. Furthermore, NOUS-209 FSMs also co-occurred with somatic mutations in MMR genes (*MSH3* and *MSH6*) and genomic stability regulators (*POLD1* and *POLE*), likely reflecting an overall increase in TMB driven by these alterations (18, 19). These mutations were frequent, with 72.6% harboring at least one pathogenic alteration in the set of four genes. Interestingly, *B2M* mutations, present in 17.8% of cases, were almost exclusively (92% of cases) found in tumors that also carried mutations in one or more of these MMR or stability genes, suggesting a relationship with the overall mutational load. However, most tumors (84.6%) with pathogenic or putative *B2M* LoF retained B2M protein expression, suggesting that these variants do not necessarily lead to complete functional inactivation. As IHC reflects posttranslational status, we prioritized it in our analysis. Thus, *B2M* mutations alone may not be sufficient to trigger immune evasion and T cell–mediated responses may still be viable. In this context, we analyzed B2M-positive colorectal cancer for correlation of the number of mutations and T-cell infiltrates. Interestingly, the intratumoral CD8^+^ cell infiltrate was correlated with the number of NOUS-209 mutation, but not with the overall TMB, supporting the selection of the mutations for the vaccine.

A distinctive feature of Lynch syndrome is the predisposition of carriers to develop multiple cancers over their lifetime (29). Strategies to prevent Lynch syndrome–associated cancers are limited. Extended colon resection reduces the risk of metachronous colorectal cancer in *MLH1*, *MSH2*, and probably even *MSH6* carriers (30). Prophylactic hysterectomy and bilateral salpingo-oophorectomy should be considered in female Lynch syndrome carriers (31). Aspirin, the only established chemopreventive agent, reduces the risk of colorectal cancer by approximately half (32). To evaluate the potential of NOUS-209 to target subsequent cancers, we divided the entire cohort into the first, second, and third malignancies. Notably, the median number of FSMs remained largely consistent regardless of chronologic order, with only a slight reduction observed in third-order tumors. This is likely to reflect the higher proportion of urothelial cancers known to carry fewer FSMs in the latter group. Additionally, cancers might be detected at earlier stages since patients are under surveillance following prior cancer diagnoses, which could influence the TMB. These findings support the potential of NOUS-209 in targeting both primary and subsequent Lynch syndrome–associated cancers.

Moreover, we were able to analyze the evolution of FSMs in a subset of nine patients with at least two consecutive tumors, an additional feature that distinguishes our study from previous studies. This longitudinal analysis allowed us to assess mutation dynamics within the context of the HLA genotype in each patient. The FSMs lost between the first and second tumors in each patient were significantly longer overall and had a higher proportion of predicted CD8^+^ T-cell strong binders compared with those kept in the second tumor. This pattern suggests a possible immune-driven selection against highly immunogenic neoantigens. Indeed, among mutations lost in patients sharing a common HLA, we identified *MSH3* in the context of HLA-A*02:01, and *TTK* and *FAHD2B* in the context of HLA-A*03:01, previously described to induce an immune response in MSI cancers (4, 17). There were three outliers in which this phenomenon did not occur. Interestingly, in two of these three outliers, a concomitant complete loss of B2M staining was observed by IHC, which likely impaired antigen presentation and could explain the simultaneous absence of selective pressure against immunogenic mutations. The ability of the immune system to shape the next tumor has already been suggested by a previous study (23), and this is further reinforced by our data.

We analyzed the repertoire of new FSMs gained in subsequent tumors to assess whether immunoediting could compromise the long-term efficacy of the NOUS-209 vaccine. The median number of gained FSMs was 34 per tumor. Importantly, the immunologic properties of the gained FSMs, specifically their cumulative peptide length and number of predicted CD8^+^ T-cell epitopes, were comparable with those of the FSMs lost after the previous tumor. Overall, these data suggest that despite immunoediting, tumors continue to acquire novel immunogenic mutations that fall within a sufficiently broad set of neoantigens encoded by NOUS-209, with the expectation that the vaccine will induce more than one specificity against the tumor of the 209 encoded acting cooperatively to fight the tumor.

Finally, we contextualized the efficacy of NOUS-209 with respect to the complete loss of B2M, which is a critical component of MHC-I antigen presentation. Complete B2M loss, which impairs the ability of neoplastic cells to present antigens and enables them to evade CD8^+^ T cell–mediated immunity, was more frequently observed in advanced-stage tumors (64.3%) than in early-stage tumors (35.7%). This finding supports the notion that delivering vaccination at an early stage before immune escape mechanisms are established is essential to maximize its clinical benefit. Overall, these findings underscore the importance of early immunologic intervention in Lynch syndrome carriers and provide a compelling rationale for the integration of broad neoantigen-targeted vaccines into interception strategies aimed at preventing cancer occurrence.

Our study has several limitations. The number of patients and tumors analyzed, particularly in the urothelial cancer subset, was small, and additional validation in larger cohorts is warranted. A second limitation is that all our findings were derived from *in silico* analyses, including mutation profiling, HLA-binding predictions, and potential neoantigen immunogenicity. Although computational approaches provide a powerful framework for evaluating neoantigen landscapes and predicting vaccine relevance, they do not account for complex *in vivo* biological variables, such as antigen processing, T-cell repertoire diversity, tumor microenvironment, and immune cell infiltration. Longitudinal immunologic data capturing T-cell responses after vaccination in patients with Lynch syndrome are needed to directly correlate antigen presence with vaccine efficacy. Third, a substantial proportion of samples lacked normal tissue reference, leading to a potential misclassification of germline mutations as somatic. We utilized population-based filters to minimize the risk, but rare germline variants might still be misclassified. However, in the context of our study, misclassification of selected common FSMs is unlikely. Fourth, although we used B2M IHC as a marker for antigen presentation, additional markers such as PD-L1 could provide a more nuanced view of immune evasion. Finally, although this study supports the rationale for immunoprevention, the clinical efficacy in reducing tumor incidence or delaying recurrence must be confirmed in prospective interventional trials.

Although our findings require validation through functional immunologic assays and future clinical studies, they strongly support the further clinical development of NOUS-209 as a promising new frontier in cancer interception in Lynch syndrome.

## Supplementary Material

Supplementary Figure S1 Supplementary Figure S1 shows the cumulative length of frameshift peptides and the estimated immunogenic potential across Lynch syndrome–associated cancers, separated by tumor type or by tumor chronology.

Supplementary Figure S2 Supplementary Figure S2 illustrates the mutational landscape of mismatch repair and genomic stability genes across tumors grouped by cancer chronology, including annotations for TMB, NOUS-209 FSMs, and tumor type.

Supplementary Figure S3 Supplementary Figure S3 shows correlations between CD8^+^ T cell infiltration (in tumor core and invasive margin) and both tumor mutational burden and NOUS-209 FSMs

Supplementary Figure S4 Supplementary Figure S4 shows the immune-driven evolution of tumor FSP profiles in Lynch syndrome–associated cancers, highlighting FSPs that are lost, maintained, or newly gained under T cell surveillance.

Supplementary Figure S5 Supplementary Figure S5 shows the number and distribution of predicted MHC-II binders among lost, kept, and gained NOUS-209–derived epitopes across patients.

Supplementary Table S1 Supplementary table S1: FSPs with high-affinity binder predictions for HLA-A*03:01 and HLA-A*02:01 recurrently lost in at least 2 patients.

## Data Availability

The code used in this study is available on GitHub at https://github.com/Nouscom/NOUS209LS-CRC-UC.git. The WES data generated in this study are available through the European Genome-Phenome Archive under accession number EGAS50000001336. Patient-related data are not publicly available because of privacy and ethical restrictions but may be made available from the corresponding author upon reasonable request and subject to institutional approval. All other raw data generated in this study are available upon request from the corresponding author.

## References

[1] Peltomäki P , Nyström M, Mecklin J-P, Seppälä TT. Lynch syndrome genetics and clinical implications. Gastroenterology 2023;164:783–99. 36706841 10.1053/j.gastro.2022.08.058

[2] Dominguez-Valentin M , Sampson JR, Seppälä TT, Ten Broeke SW, Plazzer J-P, Nakken S, . Cancer risks by gene, age, and gender in 6350 carriers of pathogenic mismatch repair variants: findings from the Prospective Lynch Syndrome Database. Genet Med Off J Am Coll Med Genet 2020;22:15–25. 10.1038/s41436-019-0596-9 PMC7371626 31337882

[3] Knudson AG Jr . Mutation and cancer: statistical study of retinoblastoma. Proc Natl Acad Sci U S A 1971;68:820–3. 5279523 10.1073/pnas.68.4.820 PMC389051

[4] Roudko V , Bozkus CC, Orfanelli T, McClain CB, Carr C, O’Donnell T, . Shared immunogenic poly-epitope frameshift mutations in microsatellite unstable tumors. Cell 2020;183:1634–49.e17. 33259803 10.1016/j.cell.2020.11.004 PMC8025604

[5] Zaidi N , Jaffee EM, Yarchoan M. Recent advances in therapeutic cancer vaccines. Nat Rev Cancer 2025;25:517–33. 40379970 10.1038/s41568-025-00820-z

[6] Leoni G , D’Alise AM, Cotugno G, Langone F, Garzia I, De Lucia M, . A genetic vaccine encoding shared cancer neoantigens to treat tumors with microsatellite instability. Cancer Res 2020;80:3972–82. 32690723 10.1158/0008-5472.CAN-20-1072

[7] Fakih M , Le DT, Pedersen KS, Shields AF, Shah MA, Mukherjee S, . First clinical and immunogenicity results including all subjects enrolled in a phase I study of Nous-209, an off-the-shelf immunotherapy, with pembrolizumab, for the treatment of tumors with a deficiency in mismatch repair/microsatellite instability (dMMR/MSI). J Clin Oncol 2022;40(Suppl 16):2515–15.35724356

[8] D’Alise AM , Brasu N, De Intinis C, Leoni G, Russo V, Langone F, . Adenoviral-based vaccine promotes neoantigen-specific CD8^+^ T cell stemness and tumor rejection. Sci Transl Med 2022;14:eabo7604. 35947675 10.1126/scitranslmed.abo7604 PMC9844517

[9] McLaren W , Gil L, Hunt SE, Riat HS, Ritchie GRS, Thormann A, . The ensembl variant effect predictor. Genome Biol 2016;17:122. 27268795 10.1186/s13059-016-0974-4 PMC4893825

[10] Karczewski KJ , Francioli LC, Tiao G, Cummings BB, Alföldi J, Wang Q, . The mutational constraint spectrum quantified from variation in 141,456 humans. Nature 2020;581:434–43. 32461654 10.1038/s41586-020-2308-7 PMC7334197

[11] Chen S , Francioli LC, Goodrich JK, Collins RL, Kanai M, Wang Q, . A genomic mutational constraint map using variation in 76,156 human genomes. Nature 2024;625:92–100. 38057664 10.1038/s41586-023-06045-0 PMC11629659

[12] Landrum MJ , Lee JM, Benson M, Brown GR, Chao C, Chitipiralla S, . ClinVar: improving access to variant interpretations and supporting evidence. Nucleic Acids Res 2018;46:D1062–7. 29165669 10.1093/nar/gkx1153 PMC5753237

[13] Li H , Durbin R. Fast and accurate short read alignment with Burrows-Wheeler transform. Bioinforma Oxf Engl 2009;25:1754–60. 10.1093/bioinformatics/btp324 PMC2705234 19451168

[14] Szolek A , Schubert B, Mohr C, Sturm M, Feldhahn M, Kohlbacher O. OptiType: precision HLA typing from next-generation sequencing data. Bioinforma Oxf Engl 2014;30:3310–6. 10.1093/bioinformatics/btu548 PMC4441069 25143287

[15] Kim Y , Ponomarenko J, Zhu Z, Tamang D, Wang P, Greenbaum J, . Immune epitope database analysis resource. Nucleic Acids Res 2012;40:W525–30. 22610854 10.1093/nar/gks438 PMC3394288

[16] Bai Y , Wang D, Fury W. PHLAT: inference of high-resolution HLA types from RNA and whole exome sequencing. Methods Mol Biol 2018;1802:193–201. 29858810 10.1007/978-1-4939-8546-3_13

[17] Bolivar AM , Duzagac F, Deng N, Reyes-Uribe L, Chang K, Wu W, . Genomic landscape of Lynch syndrome colorectal neoplasia identifies shared mutated neoantigens for immunoprevention. Gastroenterology 2024;166:787–801.e11. 38244726 10.1053/j.gastro.2024.01.016 PMC11034773

[18] Kayhanian H , Cross W, van der Horst SEM, Barmpoutis P, Lakatos E, Caravagna G, . Homopolymer switches mediate adaptive mutability in mismatch repair-deficient colorectal cancer. Nat Genet 2024;56:1420–33. 38956208 10.1038/s41588-024-01777-9 PMC11250277

[19] Mosalem O , Coston TW, Imperial R, Mauer E, Thompson C, Yilma B, . A comprehensive analysis of POLE/POLD1 genomic alterations in colorectal cancer. Oncologist 2024;29:e1224–7. 38776551 10.1093/oncolo/oyae098 PMC11379631

[20] Wang H , Liu B, Wei J. Beta2-microglobulin(B2M) in cancer immunotherapies: biological function, resistance and remedy. Cancer Lett 2021;517:96–104. 34129878 10.1016/j.canlet.2021.06.008

[21] Cornish AJ , Gruber AJ, Kinnersley B, Chubb D, Frangou A, Caravagna G, . The genomic landscape of 2,023 colorectal cancers. Nature 2024;633:127–36. 39112709 10.1038/s41586-024-07747-9 PMC11374690

[22] Maby P , Tougeron D, Hamieh M, Mlecnik B, Kora H, Bindea G, . Correlation between density of CD8^+^ T-cell infiltrate in microsatellite unstable colorectal cancers and frameshift mutations: a rationale for personalized immunotherapy. Cancer Res 2015;75:3446–55. 26060019 10.1158/0008-5472.CAN-14-3051

[23] Ballhausen A , Przybilla MJ, Jendrusch M, Haupt S, Pfaffendorf E, Seidler F, . The shared frameshift mutation landscape of microsatellite-unstable cancers suggests immunoediting during tumor evolution. Nat Commun 2020;11:4740. 32958755 10.1038/s41467-020-18514-5 PMC7506541

[24] Suzuki H , Matsumoto K, Terabe M. Ki-67 antibody labeling index in colorectal carcinoma. J Clin Gastroenterol 1992;15:317–20. 1294637 10.1097/00004836-199212000-00010

[25] Limas C , Bair R, Bernhart P, Reddy P. Proliferative activity of normal and neoplastic urothelium and its relation to epidermal growth factor and transferrin receptors. J Clin Pathol 1993;46:810–6. 8227429 10.1136/jcp.46.9.810 PMC501514

[26] Dominguez-Valentin M , Haupt S, Seppälä TT, Sampson JR, Sunde L, Bernstein I, . Mortality by age, gene and gender in carriers of pathogenic mismatch repair gene variants receiving surveillance for early cancer diagnosis and treatment: a report from the prospective Lynch syndrome database. eClinicalMedicine [Internet] 2023;58:101909. [cited 2025 Jun 1]. Available from: https://www.thelancet.com/journals/eclinm/article/PIIS2589-5370(23)00086-X/fulltext. 37181409 10.1016/j.eclinm.2023.101909 PMC10166779

[27] Møller P , Seppälä T, Bernstein I, Holinski-Feder E, Sala P, Evans DG, . Cancer incidence and survival in Lynch syndrome patients receiving colonoscopic and gynaecological surveillance: first report from the prospective Lynch syndrome database. Gut 2017;66:464–72. 26657901 10.1136/gutjnl-2015-309675 PMC5534760

[28] Seppälä TT , Latchford A, Negoi I, Sampaio Soares A, Jimenez-Rodriguez R, Sánchez-Guillén L, . European guidelines from the EHTG and ESCP for Lynch syndrome: an updated third edition of the Mallorca guidelines based on gene and gender. Br J Surg 2021;108:484–98. 34043773 10.1002/bjs.11902 PMC10364896

[29] Møller P , Seppälä T, Bernstein I, Holinski-Feder E, Sala P, Evans DG, . Incidence of and survival after subsequent cancers in carriers of pathogenic MMR variants with previous cancer: a report from the prospective Lynch syndrome database. Gut 2017;66:1657–64. 27261338 10.1136/gutjnl-2016-311403 PMC5561364

[30] Prospective Lynch Syndrome Database . Metachronous colorectal cancer risks after extended or segmental resection in MLH1, MSH2, and MSH6 Lynch syndrome: multicentre study from the Prospective Lynch Syndrome Database. Br J Surg 2025;112:znaf061. 40231433 10.1093/bjs/znaf061 PMC11997434

[31] Dominguez-Valentin M , Crosbie EJ, Engel C, Aretz S, Macrae F, Winship I, . Risk-reducing hysterectomy and bilateral salpingo-oophorectomy in female heterozygotes of pathogenic mismatch repair variants: a Prospective Lynch Syndrome Database report. Genet Med 2021;23:705–12. 33257847 10.1038/s41436-020-01029-1 PMC8026395

[32] Burn J , Sheth H, Elliott F, Reed L, Macrae F, Mecklin JP, . Cancer prevention with aspirin in hereditary colorectal cancer (Lynch syndrome), 10-year follow-up and registry-based 20-year data in the CAPP2 study: a double-blind, randomised, placebo-controlled trial. Lancet 2020;395:1855–63. 32534647 10.1016/S0140-6736(20)30366-4 PMC7294238

