# Supplementary material for: NOUS-209 Off-the-shelf Immunotherapy Has the Potential to Hit Primary and Metachronous Colorectal and Urothelial Cancers in Lynch Syndrome
Source: Mol Cancer Ther. 2025 Nov 12;25(4):650–61. doi: 10.1158/1535-7163.MCT-25-0864 (PMC13044529; doi:10.1158/1535-7163.MCT-25-0864)
Supplement: Supplementary Figure S1 — shows the cumulative length of frameshift peptides and the estimated immunogenic potential across Lynch syndrome–associated cancers, separated by tumor type or by tumor chronology. [file mct-25-0864_supplementary_figure_s1_suppsf1.pdf]

Supplementary figure S1

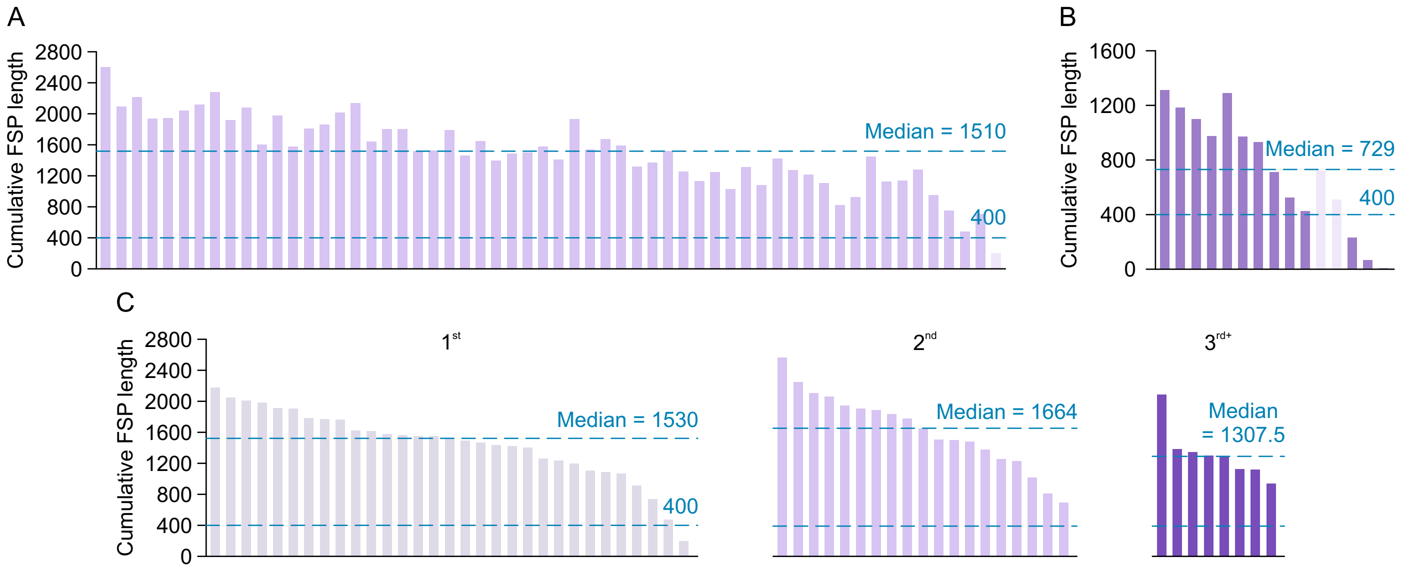

Supplementary figure S1. Cumulative length of FSPs and estimation of immunogenic potential across LS-associated cancers. Bar plots showing the cumulative length (aa) of frameshift peptides identified in the CRC cohort (A), the UC cohort (B), and the combined cohort stratified by tumor chronology—first, second or third and subsequent (C). Based on our estimate that an antigenic sequence of 400 amino acids (aa) contains on average 3 immunogenic epitopes (6), these cumulative lengths provide a proxy for the potential immunogenic burden. In panels A and B, light-colored bars represent samples classified as MSS.
