# Supplementary material for: NOUS-209 Off-the-shelf Immunotherapy Has the Potential to Hit Primary and Metachronous Colorectal and Urothelial Cancers in Lynch Syndrome
Source: Mol Cancer Ther. 2025 Nov 12;25(4):650–61. doi: 10.1158/1535-7163.MCT-25-0864 (PMC13044529; doi:10.1158/1535-7163.MCT-25-0864)
Supplement: Supplementary Figure S2 — illustrates the mutational landscape of mismatch repair and genomic stability genes across tumors grouped by cancer chronology, including annotations for TMB, NOUS-209 FSMs, and tumor type. [file mct-25-0864_supplementary_figure_s2_suppsf2.pdf]

Supplementary figure S2

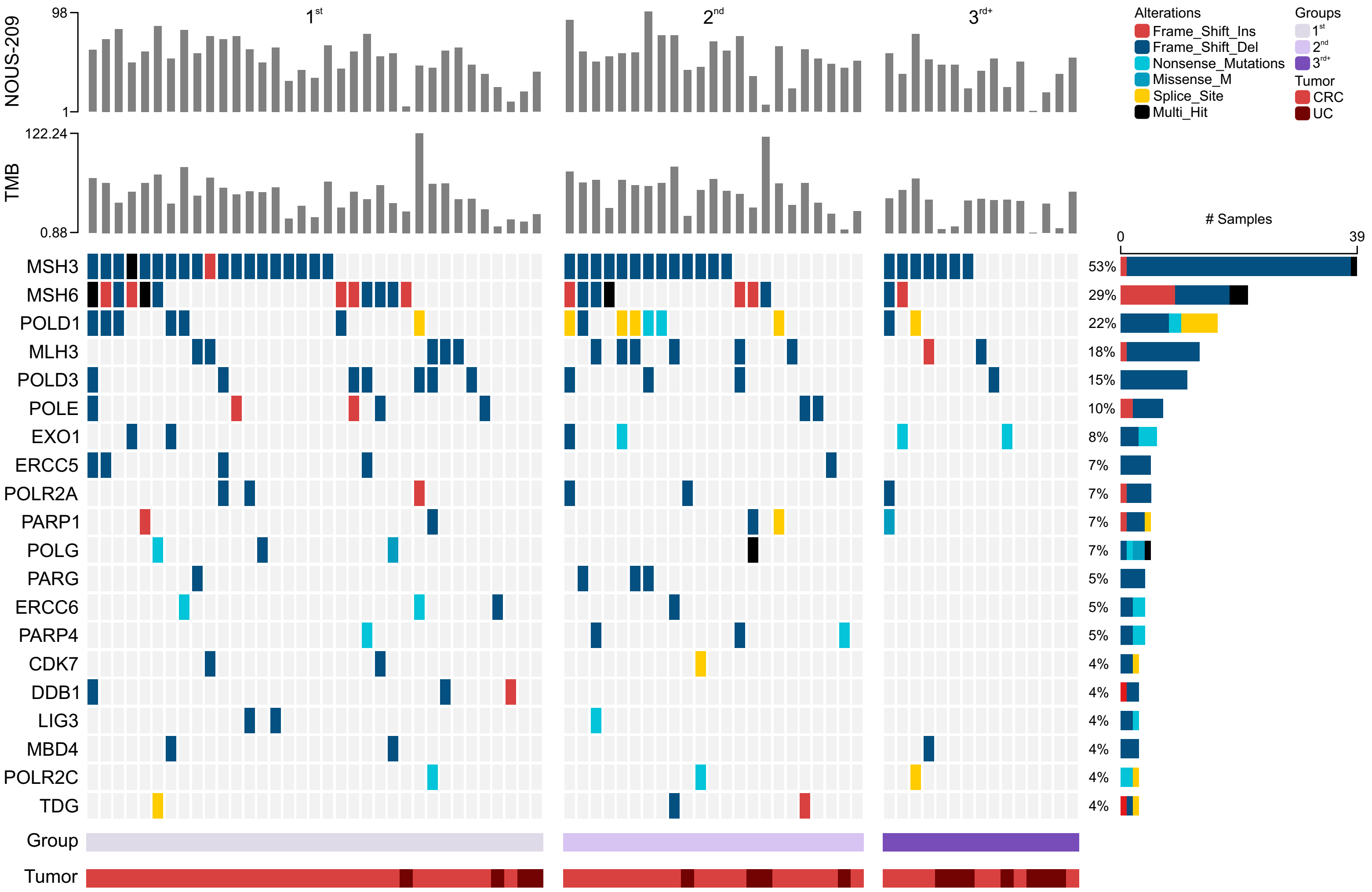

Supplementary figure S2. Systematic evaluation of somatic mutations in MMR and genomic stability pathways. Oncoplot showing the mutational landscape of mismatch, base excision, and nucleotide excision repair genes. Tumors were classified into three groups based on cancer chronology: 1st, 2nd, and $3rd^{+}$. Genes are ordered by decreasing mutation frequency. Annotations include TMB (Mut/Mb), the number of NOUS-209 FSMs, and tumor type.
