# Supplementary material for: NOUS-209 Off-the-shelf Immunotherapy Has the Potential to Hit Primary and Metachronous Colorectal and Urothelial Cancers in Lynch Syndrome
Source: Mol Cancer Ther. 2025 Nov 12;25(4):650–61. doi: 10.1158/1535-7163.MCT-25-0864 (PMC13044529; doi:10.1158/1535-7163.MCT-25-0864)
Supplement: Supplementary Figure S3 — shows correlations between CD8+ T cell infiltration (in tumor core and invasive margin) and both tumor mutational burden and NOUS-209 FSMs [file mct-25-0864_supplementary_figure_s3_suppsf3.pdf]

Supplementary figure S3

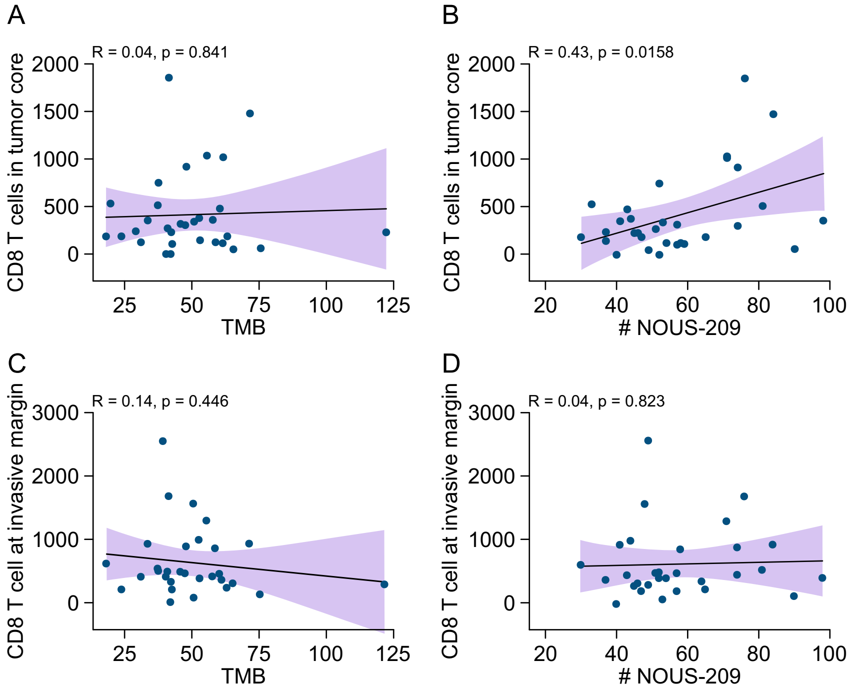

Supplementary figure S3. Scatter plot showing the correlation between CD8$^+$ T cell in tumor core with TMB (A) and NOUS-209 (B). Scatter plot showing the correlation between CD8$^+$ T cell at invasive margin with TMB (C) and NOUS-209 (D). Each point represents an individual observation (n=31). The black line indicates the fitted linear regression model with its 95% confidence interval (violet shading). Pearson's correlation coefficient (R) and p-value (p) were calculated to assess the direction and significance of the associations.
