# Supplementary material for: NOUS-209 Off-the-shelf Immunotherapy Has the Potential to Hit Primary and Metachronous Colorectal and Urothelial Cancers in Lynch Syndrome
Source: Mol Cancer Ther. 2025 Nov 12;25(4):650–61. doi: 10.1158/1535-7163.MCT-25-0864 (PMC13044529; doi:10.1158/1535-7163.MCT-25-0864)
Supplement: Supplementary Figure S4 — shows the immune-driven evolution of tumor FSP profiles in Lynch syndrome–associated cancers, highlighting FSPs that are lost, maintained, or newly gained under T cell surveillance. [file mct-25-0864_supplementary_figure_s4_suppsf4.pdf]

Supplementary figure S4

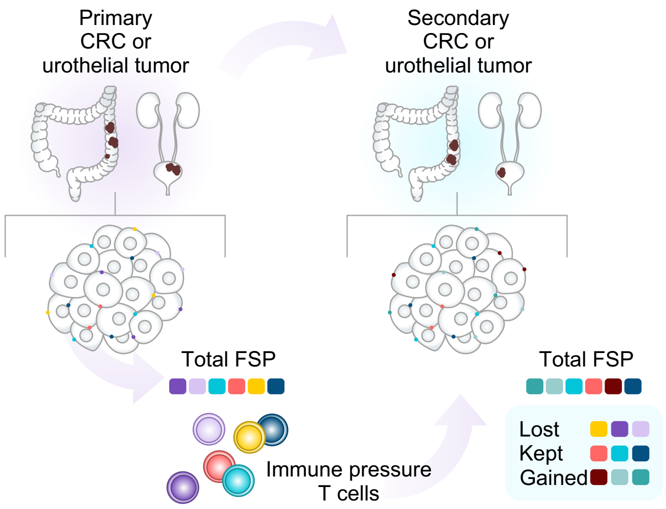

Supplementary figure S4. Evolution of tumor FSP repertoire in LS patients under immune surveillance. Schematic representation of the immune-mediated evolution of FSP profiles in LS-associated CRC or UC. The initial tumor presents a set of FSPs, which are recognized and targeted by T cells, generating immune pressure. As a secondary tumor develops, the FSP profile changes due to immune editing. Comparison of the two tumors shows FSPs that are lost, kept, or newly gained, suggesting immune editing and adaptation of tumor antigens under continuous T cell surveillance.
