# Supplementary material for: NOUS-209 Off-the-shelf Immunotherapy Has the Potential to Hit Primary and Metachronous Colorectal and Urothelial Cancers in Lynch Syndrome
Source: Mol Cancer Ther. 2025 Nov 12;25(4):650–61. doi: 10.1158/1535-7163.MCT-25-0864 (PMC13044529; doi:10.1158/1535-7163.MCT-25-0864)
Supplement: Supplementary Figure S5 — shows the number and distribution of predicted MHC-II binders among lost, kept, and gained NOUS-209–derived epitopes across patients. [file mct-25-0864_supplementary_figure_s5_suppsf5.pdf]

Supplementary figure S5

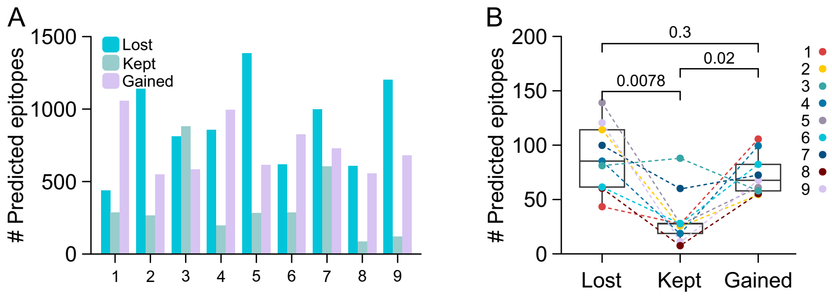


Supplementary figure S5. (A) Bar plot showing for each patient the number of MHC-II predicted binders (IC50 <500nM) for lost, kept and gained (epitopes length 12aa to 16 aa). (B) Box plot showing, for each patient, the distribution of MHC-II strong binders across NOUS-209 FSM categories (lost, kept and gained).
