# Supplementary material for: NOUS-209 Off-the-shelf Immunotherapy Has the Potential to Hit Primary and Metachronous Colorectal and Urothelial Cancers in Lynch Syndrome
Source: Mol Cancer Ther. 2025 Nov 12;25(4):650–61. doi: 10.1158/1535-7163.MCT-25-0864 (PMC13044529; doi:10.1158/1535-7163.MCT-25-0864)
Supplement: Supplementary Table S1 — FSPs with high-affinity binder predictions for HLA-A*03:01 and HLA-A*02:01 recurrently lost in at least 2 patients. [file mct-25-0864_supplementary_table_s1_suppst1.docx]

Supplementary table S1. Table of FSPs with high-affinity binder predictions for HLA-A*03:01 and HLA-A*02:01 recurrently lost in at least 2 patients.

| **HLA allele** | **FSP ID** | **Lost in (# patients)** |
| --- | --- | --- |
| HLA-A*03:01 | FSP_SLAMF1_1 | 3 |
| HLA-A*03:01 | FSP_TTK_1 | 3 |
| HLA-A*03:01 | FSP_CDC7_1 | 2 |
| HLA-A*03:01 | FSP_FAHD2B_1 | 2 |
| HLA-A*03:01 | FSP_PHACTR4_1 | 2 |
| HLA-A*03:01 | FSP_EIF2B3_1 | 2 |
| HLA-A*03:01 | FSP_EPHB2_1 | 2 |
| HLA-A*03:01 | FSP_KMT2C_1 | 2 |
| HLA-A*03:01 | FSP_CD3G_1 | 2 |
| HLA-A*03:01 | FSP_SPECC1_1 | 2 |
| HLA-A*03:01 | FSP_SMAP1_1 | 2 |
| HLA-A*03:01 | FSP_TCEB3_1 | 2 |
| HLA-A*02:01 | FSP_MSH3_1 | 3 |
| HLA-A*02:01 | FSP_KMT2C_1 | 3 |
| HLA-A*02:01 | FSP_RFC3_1 | 2 |
| HLA-A*02:01 | FSP_ROBO2_1 | 2 |
| HLA-A*02:01 | FSP_TTK_1 | 2 |
| HLA-A*02:01 | FSP_PSME4_1 | 2 |
| HLA-A*02:01 | FSP_EIF2B3_1 | 2 |
| HLA-A*02:01 | FSP_MAPRE3_1 | 2 |
| HLA-A*02:01 | FSP_SPECC1_1 | 2 |
| HLA-A*02:01 | FSP_SRPR_1 | 2 |
| HLA-A*02:01 | FSP_FXR1_1 | 2 |
| HLA-A*02:01 | FSP_XYLT2_1 | 2 |
